# Low-dose dobutamine in acute myocardial infarction with intermediate to high risk of cardiogenic shock development (the DOBERMANN-D trial): study protocol for a double-blinded, placebo-controlled, single-center, randomized clinical trial

**DOI:** 10.1186/s13063-024-08567-y

**Published:** 2024-10-30

**Authors:** Sarah Louise Duus Holle, Joakim Bo Kunkel, Christian Hassager, Redi Pecini, Sebastian Wiberg, Pernille Palm, Lene Holmvang, Lia Evi Bang, Jesper Kjærgaard, Jakob Hartvig Thomsen, Thomas Engstrøm, Jacob Eifer Møller, Jacob Thomsen Lønborg, Helle Søholm, Martin Frydland

**Affiliations:** 1 Department of Cardiology, The Heart Centre, Copenhagen University Hospital – Rigshospitalet, Blegdamsvej 2142, Copenhagen, DK-2100 Denmark; 2 Department of Clinical Medicine, Faculty of Health and Medical Sciences, University of Copenhagen, Copenhagen, Denmark; 3 Department of Cardiothoracic Anaesthesiology, The Heart Centre CopenhagenUniversity Hospital – Rigshospitalet, Copenhagen, Denmark; 4 Department of Cardiology, Bispebjerg Frederiksberg University Hospital, Copenhagen, Denmark; 5 Department of Cardiology, Odense University Hospital, Odense, Denmark; 6 Department of Cardiology, Zealand University Hospital, Roskilde, Denmark

**Keywords:** Acute myocardial infarction, Cardiogenic shock, Hemodynamic, Dobutamine, Inotropy, Neurohormonal activation, ORBI risk score, Percutaneous coronary intervention, Transthoracic echocardiography

## Abstract

**Background:**

Cardiogenic shock (CS) occurs in 5–10% of patients with acute myocardial infarction (AMI), and the condition is associated with a 30-day mortality rate of up to 50%. Most of the AMI patients are in SCAI SHOCK stage B upon hospital arrival, but some of these patients will progression through the stages to overt shock (SCAI C-E). Around one third of patients who develop CS are not in shock at the time of hospital admission. Pro-B-type natriuretic peptide (proband) is a biomarker closely related to CS development. The aim of this study is to investigate the potential for preventing progression of hemodynamic instability by early inotropic support with low-dose dobutamine infusion administrated after revascularization in AMI patients with intermediate to high risk of in-hospital CS development.

**Methods:**

This investigator-initiated, double-blinded, placebo-controlled, randomized, single-center, clinical trial will include 100 AMI patients (≥ 18 years) without CS at hospital admission and at intermediate-high risk of in-hospital CS development (ORBI risk score ≥ 10). Patients will be randomized in a 1:1 ratio to a 24 h intravenous (IV) infusion of dobutamine (5 μg/kg/min) or placebo (NaCl) administrated after acute percutaneous coronary intervention (PCI) (< 24 h from symptom onset). Blood samples are drawn at time points from study inclusion (before infusion, 12, 24, 36, and 48 h).

The primary outcome is peak plasma proBNP within 48 h after infusion as a surrogate-measure for the hemodynamic status. Hemodynamic function will be assessed pulse rate, blood pressure, and lactate within 48 h after infusion and by transthoracic echocardiography (TTE) performed after 24–48 h and at follow-up after 3 months. Markers of cardiac injury (troponin T and creatine kinase MB (CK-MB)) will be assessed.

**Discussion:**

Early inotropic support with low-dose dobutamine infusion in patients with AMI, treated with acute PCI, and at intermediate-high risk of in-hospital CS may serve as an intervention promoting hemodynamic stability and facilitating patient recovery. The effect will be assessed using proBNP as a surrogate marker of CS development, hemodynamic measurements, and TTE within the initial 48 h and repeated at a 3-month follow-up.

**Trial registration:**

The Regional Ethics Committee : H-21045751. EudraCT: 2021–002028-19. ClinicalTrials.gov: NCT05350592, Registration date: 2022-03-08. WHO Universal Trial Number: U1111-1277–8523.

## Administrative information

Note: the numbers in curly brackets in this protocol refer to the SPIRIT checklist item numbers. The order of the items was modified to group similar items (see http://www.equator-network.org/reporting-guidelines/spirit-2013-statement-defining-standard-protocol-items-for-clinical-trials/).

**Table:** 

Title {1}	**Low-dose Dobutamine in Acute Myocardial Infarction with Intermedi-ate to High Risk of Cardiogenic Shock Development (The DOBER-MANN-D trial): Study protocol for a double-blinded, placebo-controlled, single-center, randomized clinical trial**
Trial registration {2a and 2b}	Regional Ethics Committee: H-21045751. EudraCT: 2021–002028-19. ClinicalTrials.gov: NCT05350592. WHO Universal Trial Number: U1111-1277–8523
Protocol version {3}	Issue date: August 01, 2022. Protocol amendment number: 04.
Funding {4}	External financial grants: Novo Nordisk Foundation. Simon Spies Foundation. Helge Peetz og Verner Peetz og hustru Vilma Peetz Legat. Internal financial grants: Rigshospitalets Apparaturudvalg Rigshospitalets The Heart Centre’s Research Committee
Author details {5a}	Sarah Louise Duus Holle, MD Department of Cardiology The Heart Centre Copenhagen University Hospital—Rigshospitalet Ryesgade 53, 9841, DK-2100 Copenhagen, Denmark E-mail: sarah.louise.duus.holle@regionh.dk Phone: + 45 22 30 04 03
Name and contact information for the trial sponsor {5b}	Christian Hassager, MD, DMSc Department of Cardiology The Heart Centre Copenhagen University Hospital—Rigshospitalet Inge Lehmanns Vej 7, DK-2100 Copenhagen, Denmark E-mail: christian.hassager@regionh.dk. Phone: + 45 35 45 05 72
Role of sponsor {5c}	The Sponsor has status as Sponsor-Investigator and is thus directly involved with the initiation, design, and execution of the trial.

## Introduction

### Background and rationale {6a}

The prognosis for patients with acute myocardial infarction (AMI) has improved in recent years [1]. However, for patients developing cardiogenic shock (CS) related to AMI, the mortality rate is high and up to 50% [2–4]. Upon hospital admission, the majority of AMI patients present in “society for cardiovascular angiography and Interventions” (SCAI) shock stage B [5, 6]. However, a some of these patients will progress through the stages to develop overt shock (SCAI stages C–E). Approximately one third of patients who develop CS do not exhibit shock symptoms upon hospital admission [2, 3]. The mortality remains high regardless of the timing of the CS development [3]. For risk estimation of CS progression, a newly validated score “Observatoire Régional Breton sur l’Infarctus” (ORBI) risk score has been developed [7]. The risk score has a high predictive value for the development of in-hospital CS in patient with ST-elevation myocardial infarction (STEMI).

Following an AMI, elevated synthesis of the biomarker pro-B-type natriuretic peptide (proBNP) is observed, and it is released after synthesis from the cardiac ventricles as a response to stretching of the cardiomyocytes present in myocardial ischemia [8–10] and heart failure [11]. Plasma concentrations of proBNP are inversely correlated with the hemodynamic status, including cardiac output [12] and left ventricular ejection fraction (LVEF) [13]. Additionally, increased proBNP concentration has been shown to be associated with increased mortality in AMI patients [13, 14].

Dobutamine is a synthetic catecholamine with primary beta-adrenergic agonist with positive inotropic properties. The drug increases cardiac contractility and reduces systemic vascular resistance, leading to an increase in cardiac output, a decrease in left ventricular afterload, but also increases myocardial oxygen demand [15, 16]. Compromised cardiac contractility after AMI may lead to decreased cardiac output. The use of low-dose dobutamine as an inotropic cardiac support could serve as a bridge to recovery after an AMI during the potential critical period after acute percutaneous coronary intervention (PCI).

The objective of the current study is to examine, whether AMI patients with an intermediate to high risk of CS development (according to the ORBI risk score) may benefit from treatment with low-dose dobutamine as an add-on to existing standard of care therapy after acute PCI.

### Objectives {7}

#### Hypothesis

We hypothesize that early administration of inotropic support with low-dose dobutamine to patients with AMI at intermediate-high risk of in-hospital CS will decrease proBNP during the initial 48 h after randomization.

#### Primary objectives

- To evaluate the effect of dobutamine on change in peak proBNP within 48 h using blood samples taken before infusion and at 12, 24, 36, and 48 h after randomization

#### Secondary objectives

- To evaluate the effects of dobutamine on the hemodynamic function assessed by transthoracic echocardiography (TTE) performed during the first 24–48 h after infusion and at three-month follow-up
- To evaluate the effects of dobutamine on the hemodynamic function assessed by pulse rate and blood pressure within 48 h after infusion
- Changes in troponin T, lactate, and CK-MB at prespecified timepoints (before infusion, 12, 24, 36, and 48 h after infusion)
- Change in proBNP at three-month follow-up
- Change in 90-day survival rate

### Trial design {8}

This is a single-center, investigator-initiated, placebo-controlled, double-blind, randomized clinical phase II trial. Following screening in the catheterization laboratory (cath lab.) upon admission, patients with AMI and treated with acute PCI (< 24 h from symptom debut) are randomized 1:1 to receive a 24-h infusion of low-dose dobutamine or placebo.

**Fig. 1:**
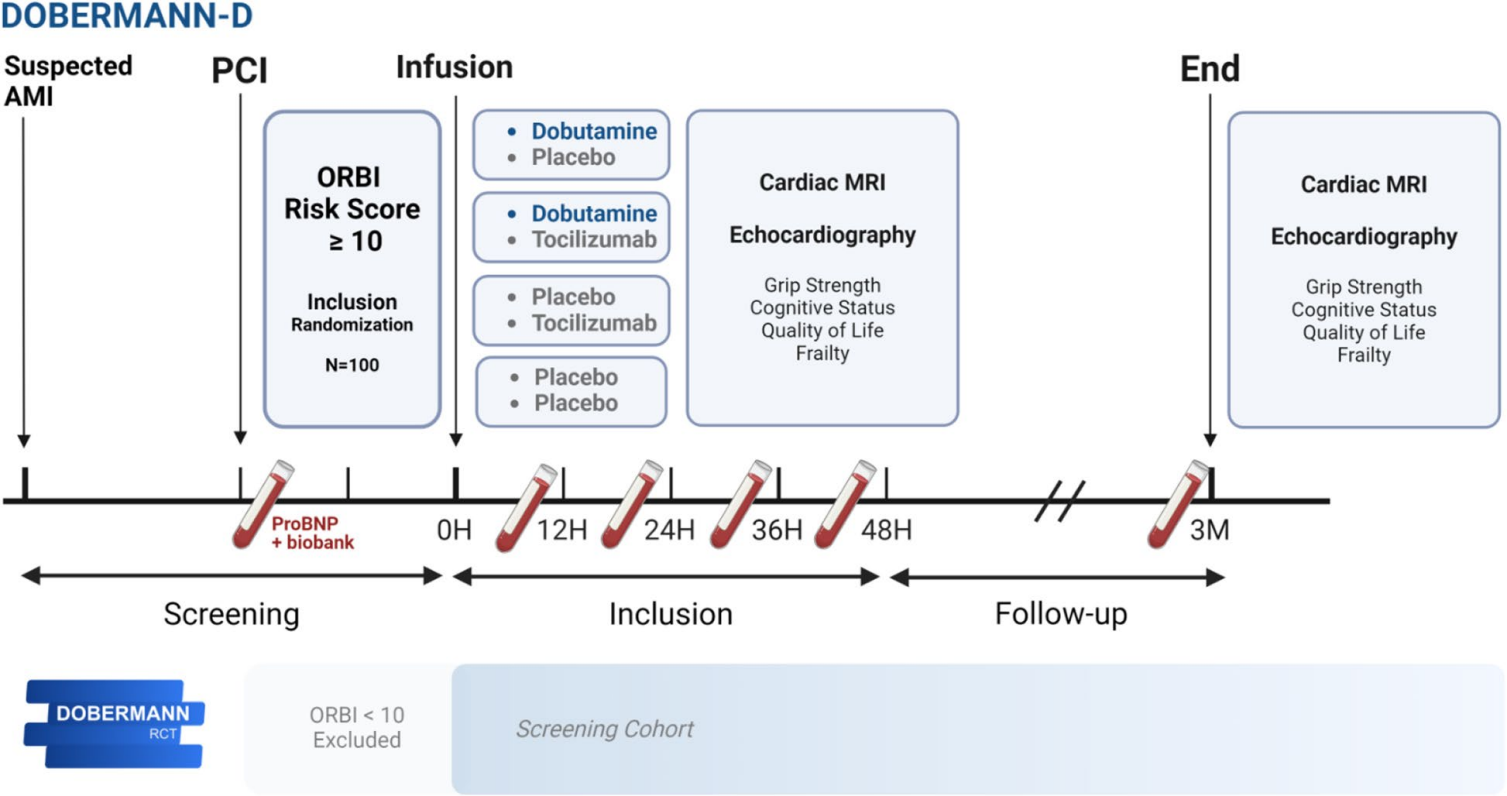
Flowchart of the DOBERMANN-D trial—from screening to follow-up. Details concerning the tocilizumab/placebo intervention group are published separately (DOBERMANN-T). Abbreviations: RCT, randomized controlled trial; AMI, acute myocardial infarction; PCI, percutaneous coronary intervention; ORBI, Observatoire Régional Breton sur l’Infarctus; MRI, magnetic resonance imaging; proBNP, pro-B-type natriuretic peptide; H, hour; M, month

**Fig. 2:**
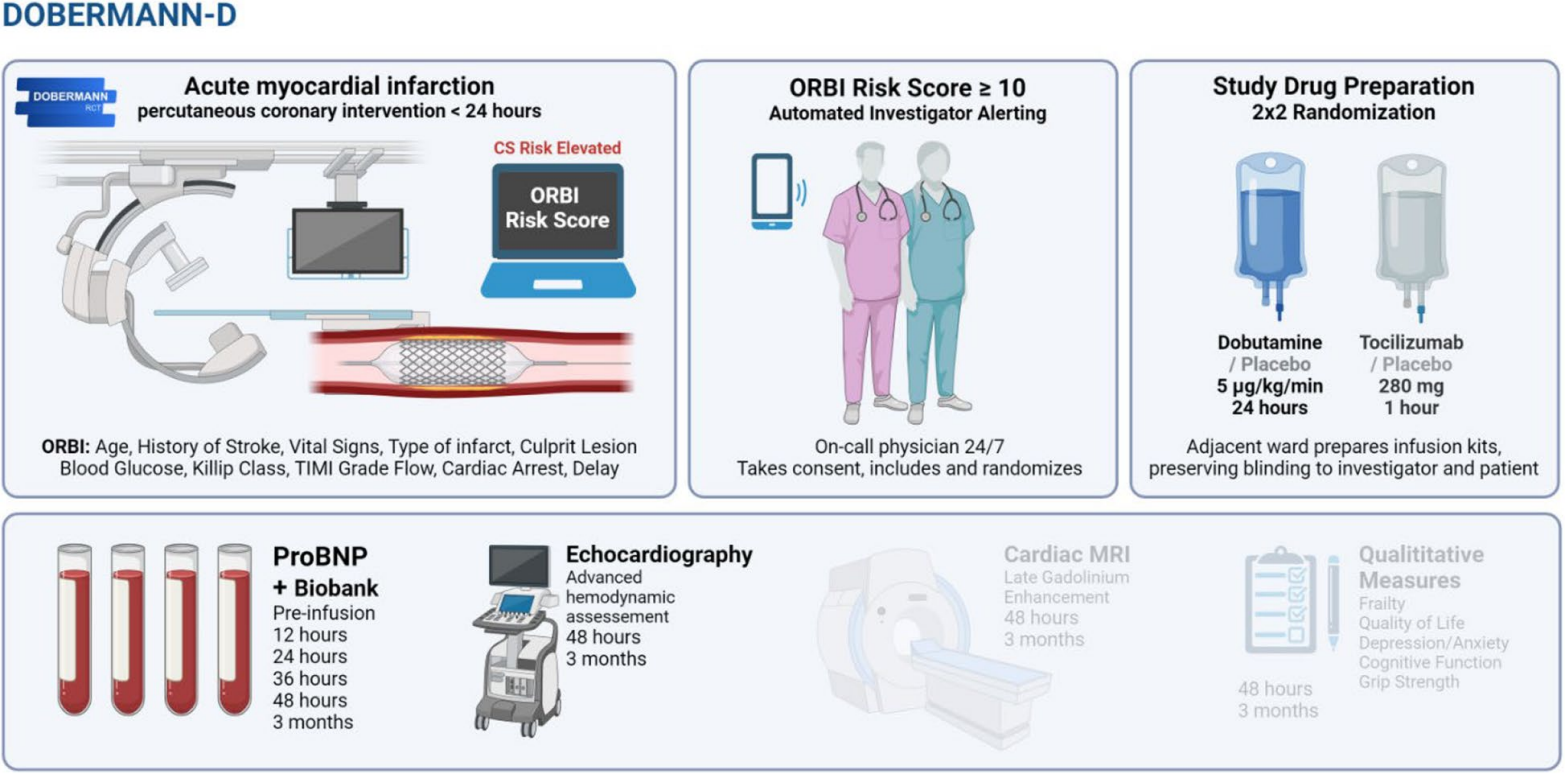
Study procedure, interventions, and endpoints. Abbreviations: CS, Cardiogenic shock, ORBI, Observatoire Régional Breton sur l’Infarctus; TIMI, thrombolysis in myocardial infarction; MRI, magnetic resonance imaging; proBNP, pro-B-type natriuretic peptide

Patients in the trial were also separately randomized to a 1-h single infusion of the interleukin 6 receptor blocker tocilizumab or placebo in a 1:1 fashion. A priori, no interaction between dobutamine and tocilizumab were expected, Figs. 1 and 2. Accordingly, the two interventions will be described as two separate trials, and the tocilizumab intervention will be described in detail in a simultaneously published manuscript (*DOBERMANN-T*).

As an explorative analysis, co-linearity between dobutamine and tocilizumab will be tested.

## Methods: participants, interventions, and outcomes

### Study setting {9}

The study is conducted at Copenhagen University Hospital Rigshospitalet in Denmark. The hospital is a public university hospital and tertiary heart center specialized in advanced cardiac care, including 24/7 acute PCI and cardiac intensive care unit (ICU) with an uptake area of 2.7 million citizens out of a total national population of 5.9 million.

Patients with AMI and symptom onset < 24 h who require acute PCI are continuously screened with the ORBI risk score in real-time in the cath lab. An automatic alert is triggered and send to the investigator on call when the ORBI risk score exceeds the predetermined threshold of ≥ 10. After enrolment in the trial, the study drug preparation is performed by the adjacent cardiac department for facilitating blinded administration of the drug/placebo by the coronary care unit (CCU).

### Eligibility criteria {10}

Inclusion criteria:

-AMI < 24 h [17]
-Urgent revascularization of culprit lesion with PCI
-Presentation within 24 h of onset of chest pain
-ORBI risk score ≥ 10 [7]
-Age ≥ 18

Exclusion criteria:

- Unwilling to give informed consent to study participation
- Unable to give consent due to a language barrier
- Comatose after cardiac arrest
- CS with systolic blood pressure < 100 mmHg for more than 30 min or the need for vasopressors to maintain blood pressure and an arterial lactate level > 2.5 mmol/L prior to leaving the cath lab
- Other major clinical noncoronary conditions (stroke, sepsis, etc.), which can explain a high ORBI risk score
- Referral for acute coronary artery bypass grafting (CABG) (< 24 h) after CAG, whereas subacute (> 24 h will be included)
- Contraindications to dobutamine infusion (sustained ventricular tachycardia prior to admission or noted in the cath lab, known pheochromocytoma, idiopathic hypertrophic subaortic stenosis)
- Pregnant or breastfeeding women
- Known, uncontrolled gastrointestinal disease predisposing to GI perforation

On all screened patients with the ORBI risk score, including eligible non-consenters, demographic and clinical data during the admission will be assessed.

### Who will take informed consent? {26a}

Participants provide consent for study involvement after receiving verbal and written information from the co-investigator. This co-investigator is either a medical doctor or a medical student supervised by a medical doctor. Patients will have the option to involve an impartial witness when they receive information about the study. The patient will be provided with a 30-min period to ask questions prior to given informed consent. The short time frame for consent has been established considering the urgency for study administration. A longer delay to administration carries the risk of CS development. This approach aligns with the Declaration of Helsinki and adheres to relevant medical research legislation for consenting adult patients.

### Additional consent provisions for collection and use of participant data and biological specimens {26b}

As part of the participation in the study, patients consent to the collection and utilization of their data and biological samples in a biobank for future analysis. These biomarkers are for research purposes only and does not have direct clinical implications. No genetic biomarkers or markers associated with the development of malignancy will be tested. Remaining biological material will be transferred to and stored for up to 10 years in a separate biobank exclusively for future research purposes. Once this 10-year period ends, blood samples will be appropriately destroyed. Patients retain the right to request the destruction of their biological specimens, in accordance with regional regulations.

## Interventions

### Explanation for the choice of comparators {6b}

To address the neurohormonal response associated with subclinical hemodynamic deterioration, dobutamine, an inotropic agent, was selected. Dobutamine increases cardiac contractility and reduces systemic vascular resistance, resulting in a decreased left ventricular afterload [15]. In dogs with experimentally induced low cardiac contractility, reduced cardiac output, and hypotension, dobutamine administration resulted in dose-dependent increase in cardiac contractility as well as output, effectively restoring arterial blood pressure [18].

The standard dosage for acute heart failure and CS typically involves an initial dose of 2–3 μg/kg/min, which can be titrated up to 20 μg/kg/min. For this trial, a low maintenance dose of 5 μg/kg/min was selected to mitigate the potential risk of tachyarrhythmias occurring in the setting of AMI [18]. No differentiation was made regarding patients treated prior to admission with beta-blockers. Furthermore, a low dose of dobutamine was chosen to minimize cardiac energy consumption and to achieve a balance of vasoconstriction and vasodilation [19].

### Intervention description {11a}

Patients will receive a 24-h IV infusion of 5 μg of dobutamine per kilogram of body weight per minute or placebo (saline). The infusion is administered as a weight-standardized preparation, mixed in isotonic saline at a rate of 5 mL per h, resulting in a total volume of 120 mL.

Individualized infusions for each patient are prepared by a collaborating cardiac unit at the center, ensuring maintenance of blinding for the treating and administering unit, including clinical staff, patients, investigators, and sponsors. Each infusion kit is labeled for safe and accurate administration. The assigned treatment arm will remain visually indistinguishable. Infusions will be administered through either a central or peripheral IV line using standard infusion pumps. The administration will be initiated as early as possible after obtaining informed consent and no later than 2 h after the PCI procedure.

### Criteria for discontinuing or modifying allocated interventions {11b}

Patients retain the right to withdraw from the study at any time and for any reason, without any consequence for the future medical care, in compliance with relevant legislation and ethical guidelines. If a patient decides to withdraw their consent to participate, the administration of the study treatment will be immediately discontinued. Data collected up to the date of withdrawal will be kept.

In certain clinical situations, the sponsor and/or the investigator may decide to withdraw a patient from the study. This decision can be made if there is a significant intercurrent illness or if the investigator or sponsor determines that termination is in the patient’s best medical interest.

Patients who withdraw may still be offered the option to continue sampling after individual agreement. An end-of-study case report form will be completed to document the patient’s final termination in the study, including an explanation for the withdrawal.

The dobutamine/placebo infusion rate may be reduced as per the protocol to 2.5 μg/kg/min (half dose) if persistent tachyarrhythmia occurs (ventricular rate ≥ 130/min for > 30 min). Additionally, the treating physician has the discretion to modify or halt the dobutamine infusion if deemed necessary. Any such changes will be recorded.

### Strategies to improve adherence to interventions {11c}

Detailed standard operating procedures (SOPs) are used to standardize procedures, and clinical personnel receive role-specific training. To ensure access to support, an investigator is available 24/7. The intervention is administered only once to minimize errors. Dose modifications are documented alongside clinical observations for safety on a case report form.

### Relevant concomitant care permitted or prohibited during the trial {11d}

Screening runs concurrently with acute patient treatment. The trial intervention supplements current standard guidelines for treatment of AMI patients [20, 21]. No delay in treatment is expected, and no restrictions apply to concomitant care or interventions. All interventions before or during admission are documented for subsequent analysis and reporting.

### Provisions for posttrial care {30}

Patients in the study are covered by the Danish Patient Compensation Scheme, like other healthcare recipients under the Danish Patient Compensation Act. After the final follow-up visit, any observed clinical or patient-reported indicators of new or worsening diseases (including mental health and depression) not currently under diagnosis or treatment will be assessed and referred if necessary.

### Outcomes {12}

#### Primary endpoint

The primary endpoint is the between-group difference (dobutamine/placebo) in peak proBNP within 48 h from randomization.

#### Secondary endpoints

- Between-group differences (dobutamine/placebo) in acute and follow-up hemodynamic function evaluated by TTE measurements
- Between-group differences (dobutamine/placebo) in pulse rate and blood pressure within 48 h from infusion
- Between-group differences (dobutamine/placebo) in proBNP at three-month follow-up
- Between-group differences (dobutamine/placebo) in troponin T, lactate, and CK-MB before infusion, 12, 24, 36, and 48 h after infusion

### Participant timeline {13}

**Table:** 

	Enrolment	Post-allocation * denotes 3-h intervals												Follow-up
**Timepoint**	**Before infusion**	**1 h**	**2 h**	**3 h**	**6 h**	*	**12 h**	*	**24 h**	*	**36 h**	*	**48 h**	**3 months**
**Enrolment**														
Eligibility screen	X													
Informed consent	X													
Randomization	X													
**Interventions**														
Dobutamine/placebo (24 h)		X												
**Assessments**														
proBNP, biomarkers	X						X		X		X		X	X
BP, P	X	X	X	X	X	X	X	X	X	X	X	X	X	
TTE													X	X
RR, SAT, Tp	X	X	X	X	X	X	X	X	X	X	X	X	X	
12-lead ECG	X						X		X		X		X	
Telemetry	X													
Biobank	X						X		X		X		X	X
CMR													X	X
QoL, MoCA, GS, frailty													X	X

*Abbreviations:* *h *hour; *proBNP *pro-B-type natriuretic peptide, *BP *blood pressure, *P *pulse rate, *TTE *transthoracic echocardiography, *RR *respiratory rate, *SAT *saturation, *Tp *temperature, *CMRI *cardiac magnetic resonance, *QoL *quality of life, *MoCA *Montreal Cognitive Assessment, *GS *grip strength

*3-h intervals

### Sample size {14}

The trial is powered towards the primary endpoint. Recent data on atrial natriuretic peptide (ANP) levels [22], measured 12 h after admission for STEMI patients, were assumed to follow a log-normal distribution with a variation coefficient of 0.59. Assuming an alpha level of 0.05, the trial will achieve a power of 0.86 to show a reduction in proBNP levels of 30% from 1338 ng/L to 937 ng/L if 88 patients is included [23].

ProBNP levels are expected to follow a log-normal distribution with a variation coefficient of 0.59. To account for dropouts and missing data, a total of 100 patients will be enrolled in the trial.

### Recruitment {15}

The recruitment period is anticipated to be three years from the enrolment of the first patient. This estimate is based on retrospective analysis of the frequencies of ORBI risk score distribution among STEMI patients and inclusion/exclusion criteria, utilizing data from prior studies conducted at the center [24].

## Assignment of interventions: allocation

### Sequence generation {16a}

This study is designed to independently evaluate the effects of the study drugs compared to placebo. The allocation sequence is generated using a computer-based pseudorandom number generator, initiated with a randomly selected seed value as input. The block sizes are predefined to ensure balance. This ensures the integrity of the allocation process and prevents any inadvertent bias.

### Concealment mechanism {16b}

Each allocation is linked with a non-sequential alphanumeric randomization key. These keys, along with their respective block variables, are securely stored within a specialized module integrated into the electronic case report form (eCRF) system, specifically Research Electronic Data Capture (REDCap).

### Implementation {16c}

Upon enrolling, the investigator will access a unique randomization key via the eCRF. The randomization key, along with the patient’s ID and weight, will be provided to the collaborating unit. The personnel responsible for drug preparation will also receive a request to prepare the study drugs as blinded infusion kits. They will use the randomization key in a secure, on-site system, temporarily revealing the assigned intervention for that specific key. After the preparation is completed, documentation of this process will be carried out using a separate unblinded eCRF. Importantly, access to this unblinded eCRF will be restricted for the investigator throughout the trial, unless stipulated by the protocol, thereby maintaining blinding integrity throughout the study.

## Assignment of interventions: blinding

### Who will be blinded {17a}

The trial is a double-blinded study, wherein the following individuals are blinded: enrolled patients, the sponsor, investigator, co-investigator, and clinical staff at the treating center.

To preserve blinding, the randomization keys are alphanumerical that do not contain information about the assigned intervention or its sequence. The keys can be communicated freely without revealing treatment details. Additionally, the prepared infusion kits are uniformly labeled and contain contents that are visually indistinguishable.

### Procedure for unblinding if needed {17b}

The unblinding process is restricted to authorized personnel responsible for study drug preparation as well as those handling emergencies and audits. Robust access controls, logging mechanisms, and comprehensive documentation of study drug preparation, including batch number registration, are enforced. A list containing information about treatment arms is securely stored in a separate system. This list is accessible solely to designated unblinded personnel, and its use is strictly governed by the protocol. For heightened security and redundancy, printed and sealed copies of the entire allocation sequence and treatment arms are kept in at least two separate physical locations. This approach ensures that recovery is feasible in the event of system malfunctions or unforeseen incidents while safeguarding the trial’s blinding integrity.

## Data collection and management

### Plans for assessment and collection of outcomes {18a}

Trained personnel, well-versed in the study protocol, are responsible for data entry and ensuring data quality. Vital parameters are retrieved from medical records. Clinical laboratory test results are rigorously validated within the Department of Clinical Biochemistry at the center, with records verifying clinical assay validity retained by the authors.

Data collection forms are digitalized within the eCRF but can also be provided in printed format for subsequent digitization. Original format copies of these forms will be preserved. Imaging data, including TTE, are stored in their raw formats in the center’s clinical imaging archiving system. The measurements are conducted and interpreted by the investigators while maintaining blinding and interobserver variability is performed. Quality of life, mental health, and cognitive assessments are administered by trained site staff and are recorded. Data are stored in the eCRF.

### Plans to promote participant retention and complete follow-up {18b}

All interventions are performed during patients’ index admission, with investigators on call 24/7/365 in case any questions or problems arise. Patients are discharged with contact information of a trial representative who can be contacted at any time during follow-up. The follow-up visit is scheduled as soon as practical to accommodate the patient’s transportation and assistance needs.

### Data management {19}

Outcome, baseline, and all other trial data are recorded using the REDCap within a hosted environment at the Capital Region of Denmark. This configuration adheres to both scientific and regulatory criteria for data entry, coding, security, and storage, with prior approval at the center for these specific purposes [25, 26]. Stringent quality control procedures are in place, incorporating double data entry and input validation for manually entered values. The study maintains a data management plan to ensure data accuracy and integrity.

### Confidentiality {27}

Within approved systems at the center, personally identifiable information is securely maintained. This includes electronic health records and related databases that covering laboratory analyses and imaging studies. The eCRF containing records of screened and included patients and is hosted by the center, overseen by the investigator in accordance with an internal data processing agreement. All data handling adheres to the European General Data Protection Regulation (GDPR) to safeguard the confidentiality and privacy of participant information.

### Plans for collection, laboratory evaluation, and storage of biological specimens for genetic or molecular analysis in this trial/future use {33}

Collected biological specimens comprise blood samples taken at baseline (after pPCI, before study intervention) and at 12, 24, 36, and 48 h during admission as well as at 3-month follow-up. Blood is collected in ethylenediaminetetraacetic acid (EDTA), lithium-heparin, sodium citrate, and serum tubes and centrifuged at 2000 g relative centrifugal force for 10 min at 20–24 °C. Samples are subsequently stored at – 80 °C. Clinical assays are performed by the center’s Department of Clinical Biochemistry and reported within the electronic health records system. Sampling is performed in accordance with a standard operating procedure for venous puncture. Biobank samples are collected for storage in a biobank by the Department of Clinical Biochemistry in accordance with a cooperation agreement.

## Statistical methods

### Statistical methods for primary and secondary outcomes {20a}

Categorical data will be reported as frequencies (number of patients), whereas continuous data will be reported as means and standard deviations (SD) for normally distributed variables or as medians and interquartile ranges (IQR, 25th–75th percentile) for variables that are not normally distributed. The alpha level is set at 0.05. Where appropriate, *p*-values and confidence intervals will be provided. Categorical outcomes will be assessed using chi-squared tests or Fisher’s exact tests, depending on the data’s characteristics. Continuous outcomes will be evaluated using the appropriate parametric or non-parametric tests based on their distribution.

The primary outcome, peak proBNP, will be log-transformed and analyzed using baseline correction (measurement before infusion) with analysis of covariance (ANCOVA) models, adjusted for the ORBI risk score and the effect of the intervention in the concurrent DOBERMANN-T trial. Interaction analyses will be conducted to evaluate the potential interaction between the two intervention groups. These models will also be applied to the secondary endpoints, which include biomarkers, vital parameters, and imaging parameters.

To mitigate the risk of type I error due to multiple comparisons, Bonferroni correction will be applied to adjust the significance levels for repeated testing across the secondary outcomes.

### Statistical software

Data will be analyzed using the latest stable version of R Statistical Software. The specific version used will be reported in the final manuscript.

### Interim analyses {21b}

No interim analyses are planned.

### Methods for additional analyses (e.g., subgroup analyses) {20b}

In a subgroup analyses, the possible interaction with the second trial intervention arm (tocilizumab/placebo) and the assigned treatment will be assessed *(DOBERMANN-T).*

### Methods in analysis to handle protocol nonadherence and any statistical methods to handle missing data {20c}

A modified intention-to-treat analysis will be performed. Missing data for the primary endpoint will be assessed, and if it exceeds 5%, the multiple imputation method will be applied.

### Plans to give access to the full protocol, participant-level data, and statistical code {31c}

A completely deidentified dataset will be made available upon reasonable request.

## Oversight and monitoring

### Composition of the coordinating center and trial steering committee {5d}

As a single-center study, daily coordination efforts are collaboratively overseen by the sponsor, primary investigator, co-investigators, and other study personnel. This center has experience in conducting clinical trials involving similar patient populations. When needed meetings are held to address operational issues and evaluate the trial’s progress.

### Composition of the data monitoring committee, its role and reporting structure {21a}

In this trial, no data monitoring committee has been established. An audit scheme is implemented, incorporating unblinded monitoring of the assigned intervention and the quality of data generated for primary endpoint analyses (see 23).

### Adverse event reporting and harms {22}

All serious adverse events (SAEs) and SUSARs are recorded by the investigators and evaluated. Each SAE and SUSAR requires the sponsor to fill in the adverse event (AE) form, including the following variables: description of event, onset and end of event, severity, relation to the intervention, action taken, and outcome. On a yearly basis, a safety report containing information on SAEs/reactions will be submitted to the Danish Health and Medicines Authority.

### Frequency and plans for auditing trial conduct {23}

Trial conduct is audited by the GCP Unit of The Capital Region of Denmark in accordance with The International Council for Harmonization of Technical Requirements for Pharmaceuticals for Human Use (ICH). GCP prior agreement that included risk analysis. The auditing process is independent and includes unblinded monitoring of treatment allocation and Investigational Medicinal Products (IMP) administration. Reports are submitted to the sponsor following each audit, which is performed at three-to-six-month intervals and covers the full data collection of at least 10% of enrolled patients.

### Plans for communicating important protocol amendments to relevant parties (e.g., trial participants, ethical committees) {25}

A digital workflow is established to ensure notification of relevant parties about protocol amendments.

Such amendments will only be implemented following regulatory approval.

### Dissemination plans {31a}

The trial results will be prepared for publication in internationally recognized peer-reviewed journals. Data from the trial will be presented at national and international conferences. Patients and their next of kin will have the opportunity to receive the trial results.

## Discussion

The DOBERMANN-D trial is an investigator-initiated, double-blinded, placebo-controlled, single-center, randomized clinical trial including AMI patients treated with acute PCI with less than 24 h from symptom onset and at intermediate to high risk of in-hospital CS development. CS is a challenge due to its variable clinical course and high mortality rate despite advances in medical therapy and revascularization techniques [2–4]. A subgroup of AMI patients is not in overt CS at hospital admission but present with subclinical markers and deteriorate hemodynamically after hours to days [2, 3]. Early identification and intervention in patients at risk of CS are important for improving outcomes.

The DOBERMANN-D trial is designed to identify AMI patients at-risk using a well-validated prediction model—the ORBI risk score [7]—and to provide early intervention through the administration of low-dose dobutamine in addition to standard therapy after acute PCI to mitigate the progression of CS. In the context of AMI, dobutamine has the potential to improve the cardiac output, which may improve organ perfusion, thereby addressing the underlying pathophysiology of CS. The improvement in myocardial performance may be particularly important in AMI patients at risk of CS development, due to a possible pre-CS stage with less pronounced signs of hemodynamic instability [2, 3].

Our hypothesis is that the administration of dobutamine prior to fulminant CS development in AMI patients at-risk could prevent the cascade of events leading to CS development, including further myocardial ischemia and pump failure. Dobutamine’s pharmacological properties with improved contractility and cardiac output could stabilize the patient hemodynamically. Increased cardiac output could improve tissue perfusion, thereby averting organ hypoperfusion and the risk of multi-organ dysfunction in the setting of CS.

Our decision to administer a low dose of dobutamine is based on considerations of safety and efficacy in order mitigate the risk of tachyarrhythmias partly based on animal studies [18, 27]. With an administration of continuous low dose of dobutamine, it is considered more likely to achieve a balance of vasoconstriction and vasodilation, thereby increasing cardiac contractility without greatly affecting peripheral resistance [19, 27]. Dobutamine as an intervention is assumed to be generally safe due to the drug’s short half-life.

This trial has been designed to detect clinical differences with the use of proBNP as a surrogate for hemodynamic instability and thereby CS development in an intermediate-high risk population. Findings of lower proBNP levels, CS development risk and/or improved cardiac function in the treatment arms would support the hypothesis that subclinical signs of CS can be routinely targeted and that this may be of benefit in an at-risk population. If this trial demonstrates a beneficial effect, it should be further tested in a larger AMI population. Additionally, research is warranted to clarify optimal dosing strategies.

To our knowledge, the DOBERMANN-D trial is the first trial to treat AMI patients at intermediate-high risk of CS development with dobutamine in a placebo-controlled setup. The use of risk stratification and targeted interventions described herein represent a step towards targeted medicine in the management of AMI.

The findings from this study have the potential to impact and optimize early risk assessment and subsequent treatment strategies in this high-risk patient population.

## Trial status

Protocol version: 2.7.6 (revision 4 from approval), 14 November 2022.

Recruitment began on 1 March 2022 and is commencing as planned, and the last patient will estimated be included by December 2024.

## Data Availability

The principal investigators will have access to the final dataset from the trial. The dataset will be stored in a secured repository with appropriate access control measures. Data will be made further available upon reasonable request at the discretion of the investigators.

## Abbreviations

AE: Adverse event
ALT: Alanine transaminase
AMI: Acute myocardial infarction
ANP: Atrial natriuretic peptide
BP: Blood pressure
CABG: Coronary artery bypass grafting
CAG: Coronary angiography
Cath lab: Catheterization laboratory
CCU: Cardiac care unit
CFS: Clinical Frailty Scale
CICU: Cardiac intensive care unit
CK-MB: Creatine kinase MB
CRP: C-reactive protein
CS: Cardiogenic shock
eCRF: Electronic case report form
EDTA: Ethylenediaminetetraacetic acid
EQ-5D-5 L: 5-level EuroQol-5
GCP: Good Clinical Practice
GCS: Glasgow Coma Scale
GI: Gastrointestinal
GS: Grip strength
H: Hours
HADS: Hospital Anxiety and Depression Scale
ICH: International Council for Harmonization of Technical Requirements for Pharmaceuticals for Human Use
IHD: Ischemic heart disease
IMP: Investigational Medicinal Products
LVEF: Left ventricular ejection fraction
MoCA: Montreal Cognitive Assessment
OHCA: Out-of-hospital cardiac arrest
ORBI: Observatoire Régional Breton sur l’Infarctus
P: Pulse rate
PCI: Percutaneous coronary intervention
proBNP: Pro-B-type natriuretic peptide
QoL: Quality of life
REDCap: Research Electronic Data Capture
RR: Respiratory rate
SAT: Saturation
SAE: Serious adverse event
SCAI: Society for Cardiovascular Angiography and Interventions
SD: Standard deviation
SIRS: Systemic inflammatory response
SOFA: Sequential organ failure assessment
STEMI: ST-elevation myocardial infarction
SUSAR: Serious adverse reaction
T: Temperature
TTE: Transthoracic echocardiography
VTI: Velocity time integral
